# Fast Melt Cocoa Butter Tablet: Effect of Waxes, Starch, and PEG 6000 on Physical Properties of the Preparation

**DOI:** 10.3390/molecules27103128

**Published:** 2022-05-13

**Authors:** Kai Bin Liew, Long Chiau Ming, Bey-Hing Goh, Kok Khiang Peh

**Affiliations:** 1 Faculty of Pharmacy, University of Cyberjaya, Cyberjaya 63000, Malaysia; liewkaibin@cybermed.edu.my; 2 PAP Rashidah Sa’adatul Bolkiah Institute of Health Sciences, Universiti Brunei Darussalam, Gadong BE1410, Brunei; 3 Biofunctional Molecule Exploratory Research Group, School of Pharmacy, Monash University Malaysia, Bandar Sunway 47500, Malaysia; goh.bey.hing@monash.edu; 4 College of Pharmaceutical Sciences, Zhejiang University, Hangzhou 310058, China; 5 School of Pharmaceutical Sciences, Universiti Sains Malaysia, Minden 11800, Malaysia

**Keywords:** fast melt tablet, cocoa butter, dapoxetine, starch, PEG 6000, wax

## Abstract

A fast melt tablet (FMT) is well regarded as an alternative delivery system that might help resolve a patient’s non-compliance issue. The main objective of this study was to develop a cocoa butter-based FMT. Additives, namely 5–15% of PEG 6000, beeswax, paraffin wax, and corn starch, were incorporated into the cocoa butter-based FMT to study the effects of these additives with the physical characteristic of a cocoa butter FMT. An optimum-based formulation was chosen according to the desired hardness and disintegration time and the taste masking property achieved with the model drug—dapoxetine. The analysis demonstrated that incorporating beeswax (15%) and paraffin wax (15%) could prolong the disintegration time by at least two-fold. On the contrary, the presence of corn starch was found to cause an increase in the hardness and reduction of the disintegration time. The disintegration mechanism might be presumed due to the synergistic effect of starch swelling and cocoa butter melting. The hardness value and in vitro disintegration time of the optimum formulation were recorded at 2.93 ± 0.22 kg and 151.67 ± 6.98 s. In terms of dissolution, 80% of dapoxetine was released within 30 min and the dissolution profile was comparable to the innovator product. The formulation was palatable and stable for at least 1 year. The exposure of the FMT formulation at 30 °C for 12 months was reported to be stable. Along with the sound palatability profile and high drug load capacity, the current formulation possesses the desired characteristics to be scaled up and marketed.

## 1. Introduction

Oral delivery is the most popular route of drug delivery [1]. Nevertheless, certain populations, especially older adults and children, experience difficulty when swallowing tablets and capsules. As a result, they tend to skip their prescribed medication, leading to non-compliance. Medication non-compliance increases healthcare costs, incurs medicine wastage, and worsens the health conditions of patients [2]. An orally disintegrating tablet (ODT), sometimes called a fast melt tablet (FMT), is a novel delivery system that holds great potential in resolving the non-compliance issue. Of note, an ODT is a patient-friendly dosage form that rapidly disintegrates when placed upon the tongue without the help of water. Thus, it offers a solution to those facing difficulty in swallowing. Currently, ODTs are manufactured using various techniques, some of these techniques are significantly time-consuming, costly, and require numerous manufacturing steps. For example, Zydis, a proprietary olanzapine ODT, is manufactured using a freeze drying technique deemed energy- and time demanding in industry settings [3]. The marketed ODT product, due to its porous structural property, possesses a fast disintegration time. However, the ODT produced is fragile and needs special packaging owing to its porous structure, increasing production costs.

Meanwhile, tablet molding is another technique usually used for ODT production. However, this technique requires a specific tableting machine, which might be deemed unaffordable by most small- and medium-sized enterprises [4]. On the other hand, a proportion of ODTs was manufactured using the sublimation technique. However, this manufacturing technique is non-feasible for thermolabile drug production; moreover, the produced ODT is fragile in nature due to its high porosity [5]. Direct compression or wet granulation by incorporating super disintegrants, as alternatives to the above-stated techniques, are perceived as potentially more robust ODT production methods, by smartly leveraging the natural characteristics of super disintegrants, drawing water into the tablet structure through swelling or wicking, with the compression leading to the formation of the tablet, with improved mechanical properties. However, there is a slightly prolonged disintegration time of approximately three minutes for this range of products.

In short, the disintegration process of marketed ODT products now focuses on producing porous structure characteristics or relying on water penetration into the tablet matrix system. Although these two concepts possess various advantages in achieving the desired outcomes, they have shortcomings, as aforementioned. Because of this situation, there is a need to explore other alternative ODT manufacturing ingredients.

Therefore, in the current research, we produced a FMT that exhibited the disintegration mechanism through the melting tablet matrix at body temperature. As the name FMT suggests, the tablet employs a tablet matrix system that consists of edible material that melts rapidly at body temperature (37 °C). For that reason, cocoa butter was used in the current study as the matrix-forming material. To complement or further improve the disintegration and hardness properties of the FMT, the effects of four additives (PEG 6000, beeswax, paraffin wax, and corn starch) were investigated. The experiment was modeled with the candidate drug dapoxetine.

## 2. Materials and Methods

### 2.1. Materials

Dapoxetine hydrochloride was a gift sample from Rakshit Drugs PVT LTD. (Gujarat, India). Kyron T-134 was a gift from Corel Pharma Chem (Gujarat, India). Cocoa butter was purchased from KL Kepong Cocoa Products Sdn. Bhd. (Selangor, Malaysia). Beeswax and paraffin wax were obtained from Apiszone Sdn. Bhd. (Melaka, Malaysia). Corn starch and polyethylene glycol (PEG 6000) were purchased from Sigma-Aldrich (St. Louis, MO, USA). Arachis oil was purchased from Panchamrut Chemicals (Mumbai, India).

### 2.2. Preparation of Cocoa Butter Based FMT

The various cocoa butter formulations are presented in Table 1. Formulation C1 was considered as a control and consists of cocoa butter only. C2–C13 were formulations with one of the additives. Four additives, namely PEG 6000, beeswax, paraffin wax, and corn starch, were used to compare their effects on the physical properties of the FMT formed. Each additive was tested at three concentrations, 5%, 10%, and 15%. A thin layer of Arachis oil was applied as a lubricant on the surface of the tablet-shaped plastic mold to ease the removal of the FMT from the mold. Cocoa butter was melted at 37 °C in a beaker. The additive (PEG 6000, beeswax, and paraffin wax) was melted at 65 °C in another beaker. The melted additive and cocoa butter were added and mixed based on the amount as stated in Table 1. Meanwhile, corn starch was added to the melted cocoa butter and stirred until homogeneous. The molten (500 mg) mixture (cocoa butter plus additive) was cast into a trapezoidal tablet-shaped PVC plastic mold individually and solidified at −20 °C for two hours. The tablet was then removed from the mold and stored in a desiccator.

### 2.3. Characterization Tests

#### 2.3.1. Evaluation of Hardness

The tablet hardness was evaluated using a TA.XT plus texture analyzer (UK) equipped with computer software, Exponent Stable Micro Systems (Ver 5.1.1.0; Stable Micro Systems Ltd., Godalming, UK). A 2 mm flat surface probe was equipped on the texture analyzer with a load of 100 g. The penetration force applied on the sample, which penetrated a 2-mm depth into the sample, was defined as the hardness of the tablet.

#### 2.3.2. Evaluation of Weight

The weight of 10 FMTs was measured using a Denver Instrument AA^®^ series AA-160 Analytical balance (Göttingen, Germany), and the mean value was reported.

#### 2.3.3. Evaluation of Thickness

The thickness of 10 FMT from each formulation was measured using a Mitutoyo micrometer (Kawasaki, Japan) at the center of the tablet.

#### 2.3.4. Friability Test

Ten FMTs were used for the friability test using a Erweka Aapparatebau-GMBH friabilator (Langen, Germany). The FMTs were weighed, and the initial total weight of ten tablets was determined by Denver Instrument AA^®^ series AA-160 Analytical balance (Göttingen, Germany). After 100 rotations at 25 rpm, the FMTs were removed from the friability tester and again weighed. Batches of tablets that lose less than 1% of the original weight are considered acceptable, according to British Pharmacopoeia 2005 [6].

#### 2.3.5. In Vitro Disintegration Time Test

The disintegration time test determines whether tablets disintegrate within a prescribed time when placed in a liquid medium under experimental conditions. The in vitro disintegration time of the FMT formulations was determined using a Pharmatest disintegration tester (Hainburg, Germany) with distilled water at 37.0 ± 0.5 °C. The disintegration time was defined as the time taken for the FMT to completely melt and pass through the screen at the bottom of each tube of the disintegration tester, such that no solid residue remained on the screen. A total of six FMTs were run for each formulation.

#### 2.3.6. In Situ Disintegration Time Test

A total of six healthy adult volunteers with ages between 22 and 55 years old participated in the study. The volunteers were briefed on the nature, purpose, duration, and risk of the study. The study protocol was approved by the University Human Research Ethics Committee (USM/JEPeM/274.3.(5)). Prior to the study, the volunteers were required to gargle their mouths with 200 mL of distilled water. One FMT was placed on the tongue of the volunteer. The volunteers were allowed to move their tongues gently while the FMT melted. The volunteers were told to spit out the test sample then rinse their mouths with 200 mL of distilled water. The time for the FMTs to melt completely in the oral cavity was measured as the in situ disintegration time.

#### 2.3.7. In Vitro Disintegration Time Test in Artificial Saliva

Artificial saliva was prepared. A total of 2 g of methyl-p-hydroxybenzoate was dissolved in 800 mL of distilled water followed by 0.625 g of KCl, 0.059 g of MgCl_2_, 0.166 g of CaCl_2_, and 1.13 g of KH_2_PO_4_. A total of 10 g of sodium carboxymethyl cellulose was dissolved in 200 mL of distilled water with the aid of heat. Both solutions were mixed and adjusted to pH 6.75 using 1 M NaOH. A Memmert water bath shaker (Schwabach, Germany) was filled with a sufficient amount of water, and the temperature was maintained at 37.0 ± 0.5 °C with 50 revolutions per minute (rpm). A total of 10 mL of artificial saliva was transferred into a 250 mL flask and kept in the water bath for 15 min for temperature equilibrium prior to the test. One FMT was put into the flask and the melting time was recorded. For each formulation, six tablets were used.

#### 2.3.8. Melting Point Test

The Perkin-Elmer Pyris 6 System DSC (Waltham, MA, USA) was used for the melting point determination. Cocoa butter, additive, and cocoa butter formulation (formulations C1, C4, C7, C10, and C13) of 5 mg were weighed in an aluminum pan and crimped. The heating rate was 10 °C/min from 0 to 100 °C under nitrogen flow (20 cm^3^/min). An empty aluminum pan was used as a reference.

#### 2.3.9. Selection of Optimum Base

An optimum base in terms of hardness and disintegration time was chosen for further testing. The formulations with in vitro disintegration time less than 120 s were shortlisted for further evaluation. Formulation with the highest hardness among the shortlisted formulations was selected as the optimum formulation.

#### 2.3.10. The Incorporation of Taste-Masked Dapoxetine in Cocoa Butter FMT Formulation

Dapoxetine was taste-masked in a previous study using Kyron T-134 at a drug:resin ratio at 1:3 (% *w*/*w*) [6]. Kyron T-134 was dispersed in a known amount of distilled water at a ratio of 1:10 (*w*/*w*). Dapoxetine HCl powder was added and the mixture was continuously stirred for 15 min. The dispersion was stirred using a magnetic bar and heated (Labinco BV LD-816 hot plate, Holland) at 60 °C for 2 h. The dispersion was filtered using Whatman filter paper. The residue on the filter paper was further dried in an oven (Carbolite, UK) at 60 °C for 12 h [6].

The optimum cocoa butter FMT formulation with in vitro DT of less than 120 s and the highest hardness were selected to incorporate taste-masked dapoxetine (120 mg) and sweetener ammonium glycyrrhizinate (30 mg). The taste-masked dapoxetine (120 mg) and ammonium glycyrrhizinate (30 mg) were added to the melted mixture of cocoa butter and additives. FMTs of 650 mg were prepared by casting molten mixture into tablet mold and solidified at −20 °C for two hours. The FMTs were then removed from the mold and stored in a desiccator until further use.

The FMT formulation was evaluated for hardness, weight, thickness, friability, in vitro disintegration time, in situ disintegration time, and in vitro disintegration time in artificial saliva.

### 2.4. Palatability Study

Twelve healthy adult volunteers between the ages of 22 and 55 years old participated in this study after providing written informed consent. Prior to the study, the volunteers were briefed on the nature, purpose, duration, and risk. The study protocol was approved by the University Human Research Ethics Committee (USM/JEPeM/274.3.(5)).

The study was divided into two phases. During the first phase, the volunteers were given the control formulation (the innovator product), followed by a wash-out period (break) of six hours before the second phase (cocoa butter FMT) proceeded. In each phase, the volunteers were required to gargle their mouths with 200 mL of distilled water prior to administration of the product. One tablet was placed on the tongue of the volunteer. The volunteers were requested to give the score based on a three-point scale to evaluate the (i) taste, (ii) after taste, (iiii) mouthfeel, (iv) irritation, and (v) acceptance of the sample. The volunteers were told to spit out the test sample, followed by rinsing their mouths with 200 mL of distilled water.

### 2.5. Content Uniformity

Ten FMTs were melted in a 100 mL volumetric flask with a solvent that was composed of 0.2 M of ammonium acetate buffer and acetonitrile, at 1:1 (% *v*/*v*), with a pH of 1.0 ± 0.1, adjusted using 3 M HCl, with gentle heating at 40 °C. The solution was subjected to sonication for 30 min. A total of 1 mL of the stock solution was drawn out and was diluted with the mobile phase to 10 mL in a volumetric flask. A 25 μL sample was injected into the HPLC system. Mean and standard deviation values were calculated.

### 2.6. HPLC-UV Assay Method

An HPLC method was developed and validated to quantify dapoxetine in the drug release study and content uniformity test. The HPLC system was comprised of a Shimadzu VP series (Japan) pump (LC-20AT vp) with a solvent cabinet, a degasser (DGU-20A_3_), a column oven (CTO-10S VP), an auto-injector (SIL-20A HT vp), a UV/VIS detector (SPD-20A vp), and computer software (LC-Solution VP). The separation was carried out using a Thermo Scientific Synchronize C-18 column (150 × 4.6 mm ID, 5 µm) (Waltham, MA, USA). The flow rate was set at 1.2 mL/min, the column temperature was set at 30 °C, and a detection wavelength of 240 nm was used. A sample of 25 µL was injected onto the column. Mobile phase A was 0.2 M of ammonium acetate buffer solution, whereas mobile phase B was acetonitrile. A time program was set. From 0.00 to 4.49 min, the system was flushed with the 50:50 mobile phase B:A (%*v*/*v*). From 4.50 to 7.99 min, the system was flushed with the 90:10 mobile phase B:A (%*v*/*v*), and the composition was immediately changed to the 50:50 mobile phase B:A (%*v*/*v*) from 8.00 to 8.01 min. The system was maintained at this ratio until 9.00 min. The method was developed and validated in a previous study [7]. The method was linear from 1 to 40 µg/mL with a correlation coefficient of 0.9994. The intraday precision and accuracy values were 0.14–1.54% and 0.63–1.83%. On the other hand, the interday precision and accuracy results were 0.49–1.83% and 1.15–1.85%.

### 2.7. Drug Release Study

The drug release studies were carried out on the optimum FMT formulation. The dissolution profile was compared to a reference product, which was the innovator product. A drug dissolution study was carried out in 900 mL of 0.1 M HCL (pH 1.0 ± 0.1) at 37.0 ± 0.5 °C, using the USP basket method at a stirring speed of 100 rpm. At pre-set time intervals of 5, 10, 15, 20, 30, 45, 60, 90, and 120 min, 1 mL of samples were withdrawn and immediately replaced with an equal volume of fresh dissolution medium. The samples were filtered through a 0.45 µm membrane filter, and the amount of drug released was determined using a validated HPLC-UV method [7]. Similarities between the dissolution profiles were assessed by a pair-wise model independent procedure, similarity factor (*f*_2_) [8,9]:

$$f_{2}=50\cdot log\left[\frac{100}{\sqrt{1+\frac{\sum_{t=1}^{t=n}{\left\{R\left(t\right)-T\left(t\right)\right\}}^{2}}{n}}}\right]$$

where *n* is the number of time points, *R(t)* is the reference profile at time point *t*, and *T(t)* is the test profile at the same time point; the value of *f*_2_ should be between 50 and 100. An *f*_2_ value of 100 suggests that the test and reference profiles are identical, and dissimilarity between release profiles increases as the value becomes smaller.

### 2.8. Stability Study

The FMTs of optimum formulation were stored in a glass container sealed with parafilm and covered with aluminum foil in desiccators containing saturated sodium chloride solution, which produced a relative humidity of 75%. The desiccators were placed inside an oven at 30 °C. After 3, 6, and 12 months of storage, the samples were removed and evaluated for physical appearance, characterization, and assay content [10].

### 2.9. Statistical Analysis

A statistical analysis was performed using Statistical Procedure for Social Science (SPSS) software (version 16, SPSS Inc., Chicago, IL, USA). The results obtained from the stability study were analyzed statistically using a one-way analysis of variance (ANOVA). When there was a statistically significant difference, a post-hoc Dunnett’s test (2-sided) was performed, compared with zero-month data. A statistically significant difference was considered at *p* < 0.05. The palatability study results were analyzed using the Mann–Whitney test. A statistically significant difference was considered at *p* < 0.05.

## 3. Results

### 3.1. Hardness

**Figure 1:**
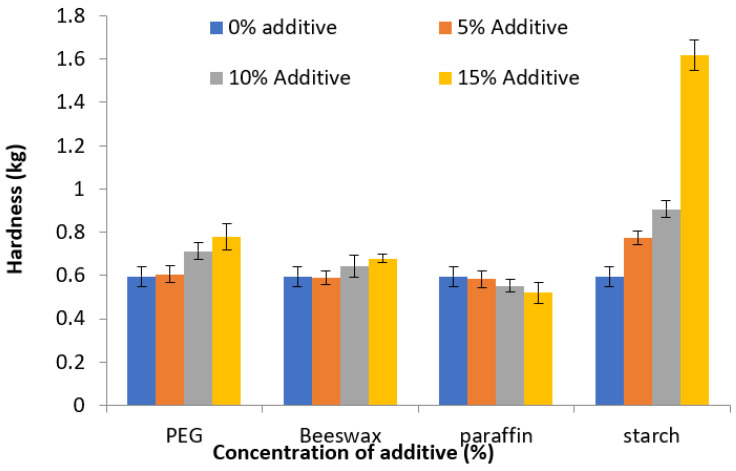
The relationship between FMT hardness and percentage of the added additive. Mean ± SD, *n* = 6.

The mean hardness and statistical analysis results of various formulations are presented in Table 2 and the relationship between hardness and concentration of each additive is presented in Figure 1. Only results of pair-wise comparisons from the post-hoc test that were statistically significant are presented in Table 2. In addition, the effect of the additive at a concentration of 15% on the hardness of cocoa butter was compared. The hardness of the various formulations ranged between 0.52 and 1.62 kg. The results showed that incorporation of PEG6000, beeswax, and corn starch, increased the hardness of cocoa butter but the incorporation of paraffin wax decreased the hardness of cocoa butter. The effect on hardness depended on the type and amount of added additive. There was a statistically significant difference in the hardness value among the various formulations.

For PEG 6000, the hardness of cocoa butter increased significantly with an increase in the amount of PEG 6000 to 10%. There was a further increase in cocoa butter hardness when PEG 6000 was increased to 15%. A mixture of cocoa butter and PEG 6000 produced tablets with higher hardness than pure cocoa butter alone. Similarly, the incorporation of beeswax increased the hardness of cocoa butter significantly but at a higher amount, of 15%. The effect was not as prominent as observed in PEG 6000. In contrast, paraffin wax at 15% reduced cocoa butter’s hardness significantly. The incorporation of corn starch from 5% to 15% increased the hardness of cocoa butter significantly. The effect of corn starch on the hardness of cocoa butter FMT was the highest among the four additives.

### 3.2. Thickness, Weight, and Friability

The mean thickness values of the various formulations were in the range of 6.09–6.17 mm. There was no statistically significant difference in the thickness values among the various formulations. The weight of the tablets of various formulations ranged between 498.70 and 503.80 mg. There was no statistically significant difference in the weight of tablets among all the formulations. All the formulations passed the friability test with less than 1% friability.

### 3.3. Disintegration Time

Two types of in vitro disintegration tests were used in the study. The in vitro disintegration time test is the general disintegration time test for general tablet formulations as per USP. The conventional in vitro disintegration time test uses 900 mL distilled water in a beaker, which is different compared to the condition in oral cavity. The in vitro disintegration time test in artificial saliva was a previously reported modified test to mimic the condition in oral cavity to test the disintegration time of ODT. A total of 10 mL of artificial saliva was added in a flask; the flask was shaken gently in a water bath maintained at 37 °C, Hence, the condition was closer to the oral cavity. The later test was conducted to verify the results of the conventional in vitro disintegration time test.

**Figure 2:**
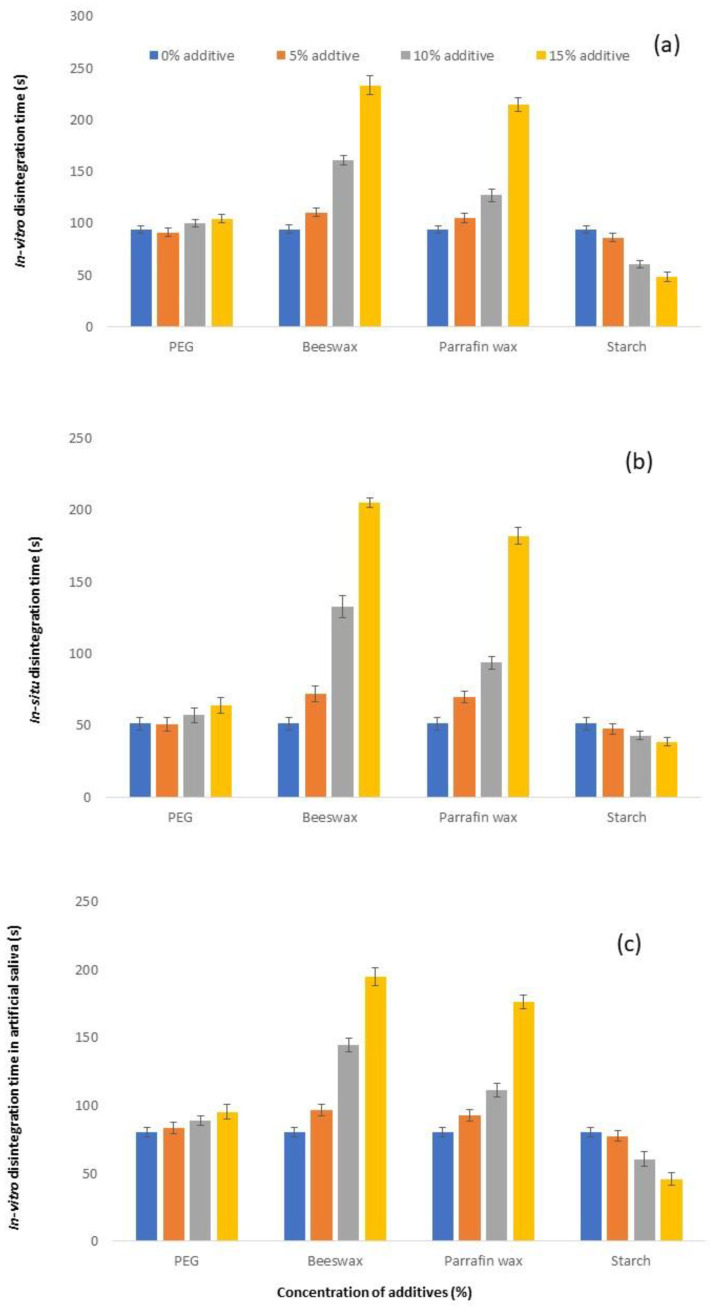
The relationship between (**a**) FMT in vitro disintegration time and the percentage of added additive; (**b**) FMT in situ disintegration time and percentage of added additive; (**c**) FMT in vitro disintegration time in artificial saliva and percentage of added additive. Mean ± SD, *n* = 6.

The relationship comparing in vitro disintegration time and the concentration of each additive, in situ disintegration time and the concentration of each additive, in vitro disintegration time in artificial saliva, and the concentration of each additive, are presented in Figure 2a–c, respectively. The in vitro disintegration time, in situ disintegration time, and in vitro disintegration time in artificial saliva, as well as the statistical analysis results of various formulations, are presented in Table 3. The in vitro and in situ disintegration times and in vitro disintegration times in artificial saliva were 48.50–233.67 s, 38.50–205.17 s, and 46.00–194.83 s, respectively. There was a statistically significant difference in the disintegration time values among the various formulations. It can be observed from Table 3 that the results of the in vitro disintegration time, in situ disintegration time, and in vitro disintegration time in artificial saliva followed a similar pattern.

There was a significant increase in the disintegration time of cocoa butter when 15% of PEG 6000 was incorporated. At a lower concentration of PEG6000, the disintegration time was not significantly affected. Similarly, the incorporation of beeswax increased the disintegration time of cocoa butter significantly, starting from 5%. A significant increase in disintegration time was observed with beeswax expansion to 10% and 15%. A similar trend was observed for paraffin wax. On the contrary, adding 10% of corn starch decreased the disintegration time significantly. The disintegration time of cocoa butter was further reduced with a further increase in corn starch content to 15%.

When the four additives were compared at a 15% concentration, there was a statistically significant difference in the disintegration time among the four additives. The increase in disintegration time was more drastic with beeswax, followed by paraffin wax and PEG 6000. Both beeswax and paraffin wax are water-insoluble hydrophobic materials. Furthermore, the melting points of beeswax (51.28 °C) and paraffin wax (43.52 °C) are comparatively higher than cocoa butter. As a result, the disintegration time of FMT formulations increased. On the other hand, as PEG 6000 is hydrophilic in nature, it could gradually disintegrate during the disintegration study. As long as the content of PEG6000 was below 15%, the disintegration time of the FMT formulation was not significantly affected. Hence, the addition of PEG6000 increased the hardness of cocoa butter without compromising on disintegration time.

### 3.4. Selection of Optimum Base

Formulation C13 was chosen as the optimum base due to the highest hardness among the formulations with a disintegration time of less than 60 s. The taste-masked dapoxetine from a previous study [6] was used as a model drug and incorporated into the final formulation as presented in Table 4. Incorporating 120 mg of the taste-masked drug and 30 mg of sweetener into the 500 mg of FMT base showed that the base could load the drug up to at least 30% of its original weight.

### 3.5. Palatability Study

The FMT formulation scored 3.0 ± 0.0 for taste, 2.8 ± 0.4 for aftertaste, 2.3 ± 0.5 for mouthfeel, 3.0 ± 0.0 for oral mucosa irritation, and 2.9 ± 0.3 for acceptance.

### 3.6. Content Determination

The dapoxetine content was within 99.89–100.98%, with a mean of 100.56 ± 1.04% in the final formulation, showing that the drug was distributed uniformly.

### 3.7. In Vitro Drug Release Study

**Figure 3:**
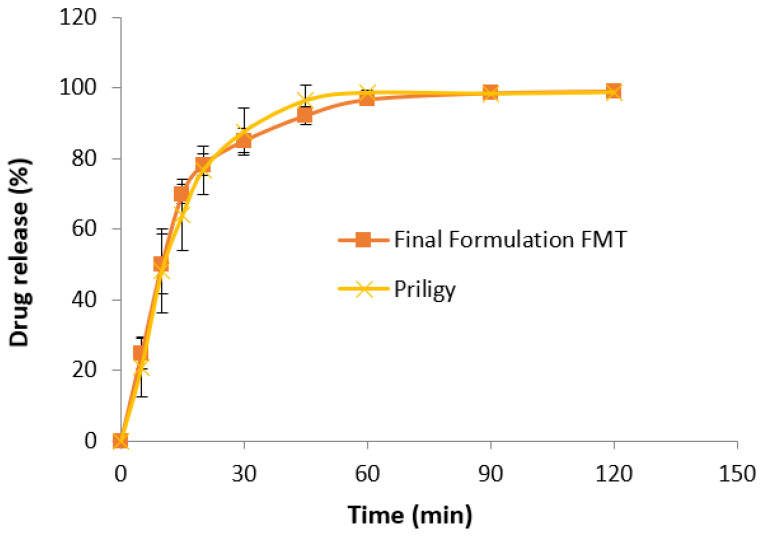
Mean dissolution profiles of the final formulation of cocoa butter-based FMT and the innovator product (Priligy) in 0.1 M HCl medium. Mean ± SD, *n* = 6.

The mean drug release profiles of the final FMT formulation and the innovator product in 0.1 M HCl medium (pH 1.0) are presented in Figure 3. The two products released more than 80% of the drugs in 30 min. The drug release profiles were closely similar with a similarity factor *f*_2_ value of 74.62. Of note, FDA has set a public standard of the *f*_2_ value between 50 and 100 to indicate similarity between two dissolution profiles.

### 3.8. Stability Study

The results of the physical properties and drug content of FMT after 12 months of storage are presented in Table 5. No statistically significant changes were observed in the physical properties and drug content (*p* > 0.05). The results showed that the final formulation was stable under experimental conditions for at least one year.

## 4. Discussion

### 4.1. Development of FMT Base

One of the ideal characteristics of FMT is fast disintegration [11]. ODT manufactured using the lyophilization [12,13,14,15], spray drying [16,17,18], and sublimation [5,19,20,21,22] method disintegrates very rapidly mainly due to the porous structure. ODT manufactured through direct compression usually relies on the effect of a superdisintegrant to draw water into the tablet matrix, hence disrupting the tablet integrity [23]. Cotton candy [11], phase transition [24], and molding technique [25] produce ODT that disintegrates mainly through the dissolution of the water-soluble matrix material. The use of a co-processed adjuvant to produce ODT was reported in the literature [26]. The current study relies on the disintegration mechanism, which is through melting off the tablet matrix at the oral cavity temperature.

Cocoa butter is commonly used as a suppository base. In the ODT formulation, it is reported as a waxy binder [27]. Cocoa butter is a fat phase material used in products such as chocolate. The crystallization and melting properties of cocoa butter are of major importance in the production of chocolate and other confectionaries. A steep melting profile with a high solid fat content at 35–37 °C, capable of being fully melted at mouth temperature, will result in glossy chocolate with good mouthfeel flavor release and easy remolding. These characteristics render cocoa butter a potential candidate to be used as the base in orally disintegrating type formulations.

Cocoa butter is a food additive that is permitted for direct addition to food for human consumption, approved by the United State-Food and Drug Administration (reference 21CFR172.861) (FDA, 2022). Cocoa butter, even though it is frequently tested in a suppository dosage form, is not well explored as an FMT tablet matrix for oral administration. Furthermore, in terms of the manufacturing process, the production of cocoa butter FMT does not require a tableting machine, which provides a lower capital expenditure commitment for enterprises adopting this manufacturing concept. As opposed to conventionally ODT manufacturing methods, the production of FMT only requires a mold and freezer, which are affordable and common in most small- and medium-sized manufacturing facilities. Hence, the method offers distinct advantages in terms of lower initial financial requirements and technical demand levels, in flowability and compressibility issues generally faced by conventional ways of ODT manufacturing.

### 4.2. Effect of Additive

In terms of strengthening or complementing the natural characteristics of cocoa butter as a matrix of FMT preparation, the effect of additives-PEG 6000, beeswax, paraffin wax, and corn starch were investigated. Incorporating additives is postulated to impact the mechanical strength and disintegration time of the FMT. Saleem et al. [28] reported that the mechanical strength of cocoa butter suppository increased with an increase in the amount of PEG 6000 incorporated in the formulation. Formulations C2–C4 (PEG 5–15%) show an increase in hardness with an increasing amount of PEG 6000 incorporated. This is in line with the finding of the previous study. Kasparaviciene et al. [29] reported that the increase in the amount of beeswax increased the hardness of the lipstick formulation. Formulations C5–C7 (beeswax 5–15%) show an increase in hardness with an increasing amount of beeswax incorporated. This is well explained as the synergistic effect of two waxes to improve the stiffness of the product formed. Beeswax can be used as a stiffening agent in formulation. Belniak et al. [30] reported that the presence of corn starch in cocoa butter suppository increased the mechanical strength of the formulation. The results from the current study are in line with the report. Formulations C11–C13 (corn starch 5–15%) showed a sharp increase in the hardness of the FMT formed.

Formulation C13 (corn starch) was selected as the optimum formulation. Corn starch was chosen because this starch has both binding and disintegrating properties [31]. It is used as a binder and disintegrant in tablet formulations [32]. Corn starch swells when it comes in contact with water, promoting disintegration [33]. A previous study reported that corn starch has a strong capillary effect of drawing water into the tablet structure and swelling upon contact with water, disrupting the tablet matrix [34]. As a result, formulations incorporated with corn starch have an advantage over other formulations because the FMT could disintegrate through two different mechanisms, namely melting of the cocoa butter matrix and swelling of corn starch, which disrupts the tablet matrix. Comparing formulation C13 (cocoa butter + 15% corn starch), it shows significant improvement in disintegration time compared to C1 (pure cocoa butter). This proves that the incorporation of corn starch into cocoa butter FMT provides a synergistic effect of combining the disintegration mechanisms of melting cocoa butter and the capillary, as well as the swelling effect of corn starch to disrupt the tablet matrix.

Beeswax and paraffin wax are both water-insoluble waxes. The melting points of beeswax and paraffin wax are 61 °C and 45 °C, respectively [35,36]. Beeswax and paraffin wax would not completely melt at 37 °C (temperature of disintegration medium and oral cavity). As a result, the disintegration time was longer. In this study, the results showed that formulations with beeswax and paraffin wax had disintegration times longer than the pure cocoa butter tablet. However, the hardness of these tablets improved in general. On the other hand, although the melting point of PEG 6000 is higher than 37 °C (MP = 60 °C), due to the hydrophilic nature of this polymer [37], the formulations containing PEG 6000 would still disintegrate, but with slightly longer times compared to the control formulation (cocoa butter base).

### 4.3. Palatability Study

Ammonium glycyrrhizinate is a unique natural plant extract product with a delightful taste [10]. It can be used in many products to sweeten and enhance the natural product flavor [38]. The Scientific Committee on Food in the European Commission has set 100 mg/day as the upper limit for regular ingestion for ammonium glycyrrhizinate [39]. A major component of success in FMT is good taste [40,41,42]. If the product does not taste good, patients and physicians will find another FMT or another appealing product [41]. FMT will disintegrate, and the active ingredient will expose directly to the taste buds on the tongue. With the incorporation of ammonium glycyrrhizinate as a sweetener, the palatability aspect of this FMT product is sound. Cocoa butter—besides having fast-melting properties—due to its chocolate taste nature, is a perfect base ingredient compared to other commonly used ODT fillers, such as lactose, microcrystalline cellulose, and inulin, which have no taste and are gritty [43]. In the palatability study, using a three-point scale, the optimum formulation recorded results in the range of 2.3–3.0 points for the five parameters, showing that the optimum formulation is well palatable and acceptable.

## 5. Conclusions

In conclusion, the fusion molding technique was shown to be feasible in preparing FMT. Formulation C13 was chosen as the optimum formulation with cocoa butter and 15% corn starch. The hardness value and in vitro disintegration times were 2.93 ± 0.22 kg and 48.50 ± 4.97 s. The tablet’s disintegration was achieved by melting the cocoa butter tablet matrix and swelling of the corn starch when in contact with saliva in the oral cavity. A total of 80% of dapoxetine was released within 30 min and the dissolution profile was comparable to the innovator product. It was palatable and stable for at least 1 year. As such, a ‘fast melt tablet’ may be a more appropriate term to describe tablets produced using the fusion molding technique than an ‘orally disintegrating tablet’.

## Figures and Tables

**Table 1:** Various cocoa butter-based formulations.

Ingredient	Formulation (mg)												
	C1	C2	C3	C4	C5	C6	C7	C8	C9	C10	C11	C12	C13
Cocoa butter	500	475	450	425	475	450	425	475	450	425	475	450	425
PEG 6000	-	25	50	75	-	-	-	-	-	-	-	-	-
Beeswax	-	-	-	-	25	50	75	-	-	-	-	-	-
Paraffin wax	-	-	-	-	-	-	-	25	50	75	-	-	-
Corn starch	-	-	-	-	-	-	-	-	-	-	25	50	75
Total	500	500	500	500	500	500	500	500	500	500	500	500	500
Additive (%)	0	5	10	15	5	10	15	5	10	15	5	10	15

**Table 2:** Hardness and statistical analysis results of various ODT formulations/additives.

Formulation	Hardness (kg)
C1 (0% additive)	0.59 ± 0.04
C2 (5% PEG6000)	0.61 ± 0.04
C3 (10% PEG6000)	0.71 ± 0.04
C4 (15% PEG6000)	0.78 ± 0.06
C5 (5% Beeswax)	0.59 ± 0.03
C6 (10% Beeswax)	0.64 ± 0.05
C7 (15% Beeswax)	0.68 ± 0.02
C8 (5% Paraffin Wax)	0.58 ± 0.04
C9 (10% Paraffin Wax)	0.55 ± 0.03
C10 (15% Paraffin Wax)	0.52 ± 0.05
C11 (5% Corn Starch)	0.77 ± 0.03
C12 (10% Corn Starch)	0.91 ± 0.04
C13 (15% Corn Starch)	1.62 ± 0.07
ANOVA	*p* < 0.05
Post-hoc test (pair-wise comparison) Additives	
PEG 6000	C1 and C3 * C1 and C4 * C2 and C3 * C2 and C4 *
Beeswax	C1 and C7 * C5 and C7 *
Paraffin wax	C1 and C10 *
Corn Starch	C1 and C11 * C1 and C12 * C1 and C13 * C11 and C12 * C11 and C13 * C12 and C13 *
15% Additive	C4 and C7 * C4 and C10 * C4 and C13 * C7 and C10 * C7 and C13 * C10 and C13 *

* *p* < 0.05.

**Table 3:** Results of in vitro disintegration time, in situ disintegration time, and in vitro disintegration time in artificial saliva of various formulations/additives.

Formulation	In Vitro Disintegration Time (s)	In Situ Disintegration Time (s)	In Vitro Disintegration Time in Artificial Saliva (s)
C1 (0% additive)	94.17 ± 3.19	51.17 ± 4.45	80.50 ± 3.73
C2 (5% PEG6000)	91.17 ± 4.07	50.83 ± 4.62	83.67 ± 4.23
C3 (10% PEG6000)	100.17 ± 3.60	57.00 ± 4.98	88.67 ± 3.56
C4 (15% PEG6000)	104.67 ± 3.78	64.17 ± 5.46	95.33 ± 5.28
C5 (5% Beeswax)	110.83 ± 4.36	71.83 ± 5.49	97.00 ± 4.29
C6 (10% Beeswax)	161.17 ± 4.36	132.67 ± 7.39	144.50 ± 5.24
C7 (15% Beeswax)	233.67 ± 9.42	205.17 ± 3.19	194.83 ± 6.88
C8 (5% Paraffin Wax)	105.50 ± 4.28	70.00 ± 4.00	92.50 ± 4.23
C9 (10% Paraffin Wax)	127.17 ± 5.64	93.83 ± 4.45	111.50 ± 4.85
C10 (15% Paraffin Wax)	215.33 ± 6.50	182.00 ± 5.83	176.33 ± 5.24
C11 (5% Corn Starch)	86.50 ± 4.37	47.50 ± 3.39	77.67 ± 3.88
C12 (10% Corn Starch)	60.67 ± 3.72	42.83 ± 2.79	60.50 ± 5.36
C13 (15% Corn Starch)	48.50 ± 4.97	38.50 ± 2.95	46.00 ± 4.52
ANOVA	*p* < 0.05	*p* < 0.05	*p* < 0.05
Additives Post-hoc test (pair-wise comparison)			
PEG6000	C1 and C4 (*p* < 0.05) C2 and C4 *	C1 and C4 * C2 and C4 *	C1 and C4 * C2 and C4 *
Beeswax	C1 and C5 * C1 and C6 * C1 and C7 * C5 and C6 * C5 and C7 * C6 and C7 *	C1 and C5 * C1 and C6 * C1 and C7 * C5 and C6 * C5 and C7 * C6 and C7 *	C1 and C5 * C1 and C6 * C1 and C7 * C5 and C6 * C5 and C7 * C6 and C7 *
Paraffin Wax	C1 and C8 * C1 and C9 * C1 and C10 * C8 and C9 * C8 and C10 * C9 and C10 *	C1 and C8 * C1 and C9 * C1 and C10 * C8 and C9 * C8 and C10 * C9 and C10 *	C1 and C8 * C1 and C9 * C1 and C10 * C8 and C9 * C8 and C10 * C9 and C10 *
Corn Starch	C1 and C12 * C1 and C13 * C11 and C12 * C11 and C13 * C12 and C13 *	C1 and C12 * C1 and C13 * C11 and C12 * C11 and C13 * C12 and C13 *	C1 and C12 * C1 and C13 * C11 and C12 * C11 and C13 * C12 and C13 *
15% Additive	C4 and C7 * C4 and C10 * C4 and C13 * C7 and C10 * C7 and C13 * C10 and C13 *	C4 and C7 * C4 and C10 * C4 and C13 * C7 and C10 * C7 and C13 * C10 and C13 *	C4 and C7 * C4 and C10 * C4 and C13 * C7 and C10 * C7 and C13 * C10 and C13 *

* *p* < 0.05.

**Table 4:** The final formulation of cocoa butter-based FMT incorporated with taste-masked dapoxetine.

Ingredient	Weight (mg/tablet)
Kyron T-134/Dapoxetine (0.75:0.25)	120
Cocoa butter	425
Corn starch	75
Ammonium glycyrrhizinate	30

**Table 5:** The results of physical properties and drug content after storage at 30 °C for 12 months.

Parameter	Time (Month)				ANOVA
	(A) 0	(B) 3	(C) 6	(D) 12	
Hardness * (kg)	2.93 ± 0.22	2.96 ± 0.24	2.96 ± 0.23	2.98 ± 0.22	*p* = 0.975
Weight * (mg)	651.20 ± 2.30	651.50 ± 1.43	651.60 ± 2.07	651.70 ± 1.89	*p* = 0.946
Thickness * (mm)	10.10 ± 0.06	10.16 ± 0.06	10.15 ± 0.05	10.12 ± 0.05	*p* = 0.060
Friability * (%)	0.35	0.36	0.34	0.35	-
In vitro disintegration time ** (s)	151.67 ± 6.98	153.00 ± 5.33	152.67 ± 3.98	154.83 ± 6.08	*p* = 0.809
In situ disintegration time ** (s)	94.33 ± 3.98	97.00 ± 3.69	94.67 ± 5.16	96.17 ± 3.97	*p* = 0.668
In vitro disintegration time in artificial saliva ** (s)	123.83 ± 4.40	124.67 ± 3.50	123.83 ± 3.82	124.83 ± 3.76	*p* = 0.951
Drug content * (%)	100.56 ± 1.04	100.11 ± 1.22	100.98 ± 0.98	99.89 ± 0.89	*p* = 0.478

* Mean of ten FMTs was used. ** Mean of six FMTs was used.
